# Lectures in the Buffalo Medical College

**Published:** 1864-10

**Authors:** 


Lectures in the Buffalo Medical College.—The preliminary and dissecting courses in this institution have already commenced, with large classes and fine prospects. The regular course of lectures commences the first Wednesday in November, and will be eminently a scientific and practical course of medical instruction, including in its curriculum, every branch of medical knowledge. The Faculty remain the same as heretofore, without change, so far as we are informed; and those who avail themselves of their instruction, will no doubt appreciate the superior advantages this institution affords.

